# Homelessness and the use of Emergency Department as a source of healthcare: a systematic review

**DOI:** 10.1186/s12245-022-00435-3

**Published:** 2022-07-28

**Authors:** Neha Vohra, Vibhu Paudyal, Malcolm J. Price

**Affiliations:** 1 College of Medical and Dental Sciences, University of Birmingham, Edgbaston, Birmingham, B15 2TT UK; 2 Institute of Applied Health Research, University of Birmingham, Birmingham, UK; 3 NIHR Birmingham Biomedical Research Centre, University Hospitals Birmingham NHS Foundation Trust, Birmingham, UK

**Keywords:** Homelessness, Emergency department, Health disparity

## Abstract

**Background:**

Persons experiencing homelessness (PEH) often use hospital Emergency Department (ED) as the only source of healthcare. The aim of this study was to undertake a systematic review to identify the prevalence, clinical reasons and outcomes in relation to ED visits by PEH.

**Methods:**

A protocol-led (CRD42020189263) systematic review was conducted using search of MEDLINE, EMBASE, CINAHL and Google Scholar databases. Studies that reported either the prevalence of homelessness in the ED or clinical reasons for presentation to ED by PEH and published in English language were included. Definitions of homelessness used by study authors were accepted.

**Results:**

From the screening of 1349 unique titles, a total of 36 studies were included. Wide variations in the prevalence and key cause of presentations were identified across the studies often linked to differences in country, study setting, disease classification and data collection methods. The proportion of ED visits contributed by PEH ranged from 0.41 to 19.6%. PEH made an average of 0.72 visits to 5.8 visits per person per year in the ED [rate ratio compared to non-homeless 1.63 to 18.75]. Up to a third and quarter of the visits were contributed by alcohol-related diagnoses and substance poisoning respectively. The percentage of PEH who died in the ED ranged from 0.1 to 0.5%.

**Conclusions:**

Drug-, alcohol- and injury-related presentations dominate the ED visits by PEH. Wide variations in the data were observed in regard to attendance and treatment outcomes. There is a need for prevention actions in the community, integrated discharge and referral pathways between health, housing and social care to minimise frequent usage and improve attendance outcomes.

**Supplementary Information:**

The online version contains supplementary material available at 10.1186/s12245-022-00435-3.

## Introduction

The global prevalence of homelessness is estimated to be around 2%, with approximately 150 million people experiencing homelessness. Additionally, 20% of the world’s population are estimated to lack adequate housing [1]. The definition of homelessness differs between countries. The US Department of Housing and Urban Development (HUD) defines homelessness as the lack of a fixed, regular and adequate night-time residence [2]. In the UK, the statutory definition of homelessness includes those living in temporary shelters, hostels and squats; street dwellers or those living (sofa surfing) in family and friends’ houses; and those who currently have an accommodation but are not able to ‘reasonably occupy’ it due to threat of eviction or violence [3, 4]. Homelessness has been on the rise in industrial economies and particularly those street dwelling in urban areas since the 2010 global recession. In the USA, it is known that approximately 1.5 million people experience homelessness every year [5]. In England, over 200,000 households experience homelessness every year [6].

Statistics show that approximately 25% of persons experiencing homelessness (PEH) have a diagnosis of at least one serious mental illness. These include bipolar disorder, schizophrenia, major depression and post-traumatic stress disorder [7]. Multi-morbidity, defined as the presence of multiple, simultaneous, chronic conditions, is also highly prevalent in PEH [8]. The average life expectancy among the homeless population in the USA is a mere 48 years [7], and in the UK, the mean age at death is 45 years for males and 43 years for females [9]. Cardiovascular health conditions, drug overdose and accidents have been recorded as contributing factors to the higher mortality rates seen in this community [9].

There remain important disparities in access to health between PEH and non-homeless populations. One US study reported that one in four homeless respondents had been unable to access medical care when they required it [10]. In England, PEH are approximately 40 times less likely to be registered with a mainstream general practice than non-homeless persons [11]. Physical and mental inability to navigate services, healthcare costs and perceived stigma surrounding PEH when accessing these services have been shown to be significant barriers to accessing primary healthcare. These barriers to accessing primary healthcare and substance misuse services are known to contribute to higher rates of utilisation of the emergency department (ED) by PEH [8, 12]. The ED, however, represents a high cost and resource intense environment, making it challenging for healthcare professionals to care for PEH who often have a multitude of diagnosed and undiagnosed health conditions, in addition to poor social circumstances. It is imperative that service providers are acquainted with up-to-date evidence in relation to homelessness and its relationship with causes, pattern, frequency and outcomes of ED presentations. Comparison of PEH data with the general population can enable identification of the extent of disparity in access and outcomes.

Currently, there lacks a comprehensive systematic review which incorporates the range of literature on patient experience of homelessness and its link to the utilisation of ED for healthcare. PEH often frequent urban areas and streets and many are known to use the ED as their only source of healthcare. In particular, the prevalence of homelessness among users of the ED, frequency of (repeat) visits to the ED by PEH, primary reasons for presentation and mortality outcomes of PEH in the ED have not been synthesised using systematic review methodology. The aim of this study is to undertake a systematic review to identify the prevalence of ED visits made by PEH, primary reasons for presentation to the ED and associated prevalence, and mortality (deaths) of PEH in the ED. This study will also aim to compare the data with non-homeless populations from the same study setting where available.

## Methods

### Study design and method

This study was conducted according to the PRISMA (Preferred Reporting Items for Systematic Review and Meta-Analyses) guideline (Additional file 1). A protocol was registered with PROSPERO (CRD42020189263).

### Data source and selection process

A systematic search of the literature was undertaken in MEDLINE, Embase, CINAHL and Google Scholar databases published between 2009 and October 2020. The key search terms and medical subject headings included homelessness, homeless persons, emergency department, accident and emergency (Additional file 2).

### Inclusion and exclusion criteria

Studies were included if they were primary research studies of any design, including prospective observational studies, retrospective database review and interventional studies that reported either the prevalence of homelessness in persons who present to the ED or reasons for presentation to ED by PEH, and published in English language. The definitions of homelessness used by study authors were accepted for the purpose of the review.

### Study selection

All stages of the screening and selection process were carried out according to the inclusion and exclusion criteria. Title and abstract screening were followed by full-text screening. Two reviewers (NV and VP) undertook the screening.

### Data extraction and quality assessment

The data extraction form was developed based on the review’s aims and objectives. The tool was refined, reviewed and piloted before use. The following information was extracted: study author(s), study year, study country, study aims, study design and duration, setting and study population, number and/or proportion of unique patients from the study populations identified as PEH, number and/or proportion of visits to the ED contributed by PEH, primary reason for presentation to the ED by PEH including number and proportions, mean number of ED visits per person per year, and deaths of PEH in the ED. Data were also extracted for non-homeless populations from the same study setting where available for the purpose of comparison.

Quality assessment of the included studies was conducted by two authors (NV and VP) using an adapted tool developed to assess quality of prevalence studies [13]. The tool consisted of 10 risk of bias items and included quality criteria referring to the target population representativeness, non-response bias, appropriateness of numerator(s) and denominator(s) for the parameters and summary of the overall risk of bias. The summary of the overall risk gives each study a total score from 0 to 9 which classifies each study into either low risk of bias (0–3 points), moderate risk of bias (4–6 points) or high risk of bias (7–9 points) [13].

### Data synthesis and analysis

Where sufficient data were reported, the prevalence of homelessness among the ED attendees was calculated for each study in two ways: (a) the number of unique PEH attending the ED was divided by the total number of unique persons attending the ED during the study period and (b) the number of ED visits by PEH was divided by the total number of ED visits during the study period.

Meta-analysis was planned for the following category of data including the prevalence of presentations to the ED contributed by PEH, the primary reasons for presentation to the ED (%), the mean number of visits to the ED by homeless persons, per person, per year [14], and the number of deaths of homeless persons in the ED. However, due to the high levels of heterogeneity, it was decided that meta-analysis was not appropriate.

A number of studies reported the mean number of ED visits but few reported the standard deviation. We used the mean number of ED visits in each group together with the study follow-up period to calculate the mean yearly attendance rates. We then calculated the log rate ratio and its standard error assuming a Poisson distribution for the rate in each group. These were then exponentiated.

## Results

**Fig. 1:**
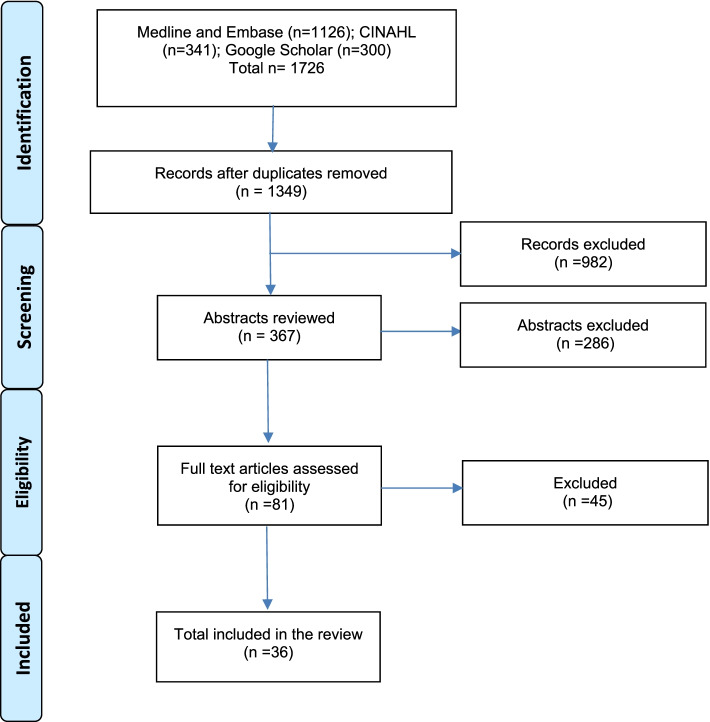
PRISMA flowchart

The electronic searches returned a total of 1726 records, from which 1349 unique titles were screened for full texts, of which 36 studies [15–50] fulfilled the eligibility criteria and were included (Fig. 1). The majority of these studies were published in the USA (*n* = 27), followed by Australia (*n* = 4), the UK (*n* = 1), Canada (*n* = 1), Turkey (*n* = 1), Ireland (*n* = 1) and Finland (*n* = 1) (Table 1).

**Table 1:** Reviewed literature on homelessness in the ED

	Author(s), year	Study year	Country	Study aim	Study design and study duration	Setting and study population	Number and/or proportion of unique patients who are homeless	Number and/or proportion of ED visits made by homeless persons	Key reasons for presentation to the ED	Mean number of ED visits per person, per year
1	Tadros et al. 2016 [15]	2016	USA	To analyse changes in ED utilisation of homeless patients and compare that with non-homeless visits	Comparative analysis of the 2005 and 2010 NHAMCS dataset	Patients presenting to non-federal hospital ED and outpatient departments		In 2010, 679,854 (0.55%) out of 124,043,357 presentations to the ED were made by homeless persons		In 2010, homeless persons made an average of 5.8 visits per person per year compared with 1.7 by non-homeless persons
2	Oates et al. 2009 [16]	2009	USA	To analyse the national utilisation of the ED by the homeless population	Cross-sectional, secondary analysis of data using NHAMCS dataset	Patients presenting to non-federal hospital ED and outpatient departments		In 2005, 472,922 (0.41%) out of 115,322,815 presentations to the ED were made by homeless persons		
3	Holtyn et al. 2017 [17]	2017	USA	To examine the relationship between ED utilisation and alcohol use in homeless alcohol-dependent adults	Analysis of self-report assessments of alcohol and emergency department use alongside random breath collections	Homeless, alcohol-dependent (met DSM-IV criteria) adults from an inpatient detoxification unit and homeless community agencies			Out of 86 recorded ED visits, 29.1% presented due to alcohol intoxication, 4.7% for alcohol withdrawal, 2.3% for drug/medication overdose, 11.6% for psychological problems and 18.6% for medical problems	Average of 4.4 ED visits per person per year
4	Brown et al. 2010 [18]	2010	UK	To determine whether the rate of attendance was related to the outside temperature	A retrospective study of routine ED computer records from 2003 to 2008	Patients presenting to the Northern General Hospital ED and data from the Weston Park Weather Station		2930 (0.55%) out of 528,573 visits to the ED were made by persons identified as homeless		
5	Cheung et al. 2015 [19]	2015	Canada	To examine the relationship between ED use and substance dependence among homeless individuals with concurrent mental illness who participated in a ‘Housing First’ intervention trial	Analysis of administrative data and findings from Vancouver At Home survey data	Homeless or precariously housed individuals who met criteria for a mental disorder with or without concurrent substance use dependence; administrative data collected from six urban hospitals in the Vancouver Coastal Health Authority				Average of 2.1 ED visits per person, per year
6	Brown et al. 2013 [20]	2013	USA	To compare the ED visit characteristics of younger homeless adults with those of older homeless adults	Analysis of a systematic random sample of ED visits using NHAMCS dataset from 2005 to 2009	Patients presenting to non-federal hospital ED and outpatient departments		2,808,000 (0.6%) out of 468,000,000 ED visits were made by homeless adults	Psychiatric issues were more frequent in younger than older homeless adults (23% vs 15%; *P* = 0.01). Older homeless adults were more likely to suffer injuries (28% vs 21%; *P* = .04) and cardiovascular complaints (11% vs 5%; *P* = 0.02) than younger homeless adults	
7	Raven et al. 2017 [21]	2017	USA	To identify ED use patterns and factors associated with ED use in adults 50 and older	Initial screen for study eligibility followed by analysis of baseline interview and medical records	Random sample of adults aged 50 years and older from homeless encampments, recycling centres, overnight homeless shelters and meal programmes			Out of 348 recorded visits to the ED, 23.9% presented for chronic illness, 21.6% for new illness, 19.2% for pain, 16.4% for injury, 8.3% for analgesic requirement and 5.8% for mental health issues	
8	Ku et al. 2010 [22]	2010	USA	To assess whether homelessness or associated characteristics independently predicted ED use	Descriptive, cross-sectional secondary analysis of ED visits using NHAMCS dataset for the years 2005 and 2006	Patients presenting to non-federal hospital ED and outpatient departments		1.1 million (0.5%) out of 234 million weighted ED attendances during the 2-year time frame were by homeless patients	Out of 550,000 recorded visits to the ED, 304,000 (55.3%) patients presented due to injuries, 100,000 (18.3%) due to alcohol or other drug use, 57,000 (10.4%) due to psychiatric diagnoses and 38,500 (7%) due to respiratory diagnoses	Average of 0.72 ED visits per person, per year
9	Feldman et al. 2017 [23]	2017	USA	To explore whether prevalence of homelessness in the ED varied between weekdays and weekends and between seasons	Prospective, 5-question homelessness screening survey of eligible participants attending the ED between May 2015 and February 2016	Patients, who are not critically ill, registered with 3 EDs in north-eastern Pennsylvania	309 (7.03%) out of 4395 participants were cited as experiencing homelessness			
10	Jackson et al. 2019 [24]	2019	USA	To describe demographics and proportion of ED patients who have experienced homelessness	Cross-sectional survey of a convenience sample of patients presenting to the ED from September to December 2016	Patients presenting to Urban Atlanta ED	475 (51.5%) out of 923 ED patients who completed the survey stated some degree of homelessness in the previous year			
11	Lee et al. 2019 [25]	2019	Australia	To compare the prevalence of homelessness in consecutive patients presenting to the ED	Prospective screening of housing status and retrospective audit of administrative data for patients presenting to the ED during a 7-day period in 2017	Patients presenting to an inner metropolitan hospital ED in Melbourne Sample size: 1275 ED presentations involving 1208 individual patients (7-day period)	40 (7.9%) of the 504 prospectively screened patients were identified as homeless and 16 (2.3%) of the 704 non-screened patients were identified as homeless			
12	Tsai et al. 2013 (a) [26]	2013	USA	To examine the proportion of homeless veterans among users of Veteran Affairs EDs and compare homeless and housed VA ED users’ clinical characteristics	Cross-sectional study analysing national VA ED user’s administrative data from the fiscal year 2010	Homeless veterans presenting to VA EDs		64,091 (6.89%) VA ED users identified as homeless out of 930,712 veterans that utilised VA EDs	Out of 64,091 recorded visits to the ED, 13.55% presented for alcohol disorder, 11.92% for drug disorder, 37.72% for psychiatric diagnoses (35.68% for non-substance misuse related), 12.84% for any pain diagnosis, 26.30% for congestive heart failure and 7.49% for chronic pulmonary disease	Average of 3.38 (SD=4.01) ED visits per person per year compared with 2.07 (SD=1.09) for non-homeless users
13	Rodriguez et al. 2009 [27]	2009	USA	To determine the extent that people experiencing homeless present to the ED for social issues	Prospective case-control study conducting interviews between July 2006 and March 2007	Patients in the treatment areas of one urban hospital ED		9806 (19.5%) out of 50,172 visits to the ED in 2006	Out of 191 homeless patients, 29% stated that hunger, safety and lack of shelter were the primary reasons for presenting to the ED	Average of 5.8 ED visits per person, per year (SD= 2.2)
14	Lin et al. 2015 [28]	2015	USA	To determine which factors are associated with frequent ED visits and hospitalisations among the insured homeless population	Retrospective, cross-sectional study using BHCHP electronic database from January to December 2010	Homeless Medicaid recipients who received service from BHCHP			Out of 25,771 recorded visits to the ED, 15.2% of patients presented for alcohol-related disorders, 7.6% for psychiatric disorders (not including substance misuse-related conditions), 5.3% for drug-related disorders, 14% for injury and poisoning, 7% for respiratory disorders and 5% for circulatory disorders	Average of 3.97 ED visits per person, per year
15	Mackelprang et al. 2014 [29]	2014	USA	To describe injury characteristics and circumstances among individuals identified as homeless in the ED	Cross-sectional, case-control study using the NEISS database between January 2007 and December 2011	Patients with product-related injuries who presented to NEISS EDs	268 (0.0142%) out of 1,885,274 unique cases that presented to NEISS ED’s with product-related injuries involved a homeless person		Out of 268 recorded visits to the ED, 13.8% had alcohol involvement and 3.4% had drug/substance use involvement	
16	Doran et al. 2016 [30]	2016	USA	To quantify the presence of housing instability, homelessness, and other selected social determinants of health in ED patients	Cross-sectional survey of a random sample of ED patients from June to August 2014	Patients presenting to an urban public hospital ED		Out of 625 visits to the ED, 19.6% reported homelessness or lack of stable housing in the past 2 months		
17	Moore et al. 2011 [31]	2011	Australia	To describe patterns of service use and predict risk factors for re-presentation to an ED among homeless persons	Retrospective analysis using computerised patient administration system from January 2003 to December 2004	Patients presenting to a principal referral hospital ED	1595 (3.9%) out of 40,942 individual patients were homeless	6689 (10.4%) out of 64,177 visits to the ED were made by the homeless population		Average of 2.1 ED visits per person, per year
18	Hammig et al. 2014 [32]	2014	USA	To determine the clinical characteristics of homeless patients presenting to the ED, focusing on unintentional and intentional injury events and related comorbid conditions	Retrospective cohort study analysing ED visits from the NHAMCS database from 2007 to 2010	Patients presenting to non-federal hospital ED and outpatient departments		603,000 (0.5%) out of 119,993,000 visits to the ED annually were made by homeless patients	Out of 603,000 reorded visits to the ED, 55% were injury related and 45% were non-injury related	
19	Mackelprang et al. 2015 [33]	2015	USA	To analyse the prevalence and characteristics of ED and inpatient admissions among homeless and unstably housed youth	Retrospective cohort study using electronic medical records from July 2009 to June 2012	Patients presenting to the ED or inpatient departments of two urban teaching hospitals			Out of 1151 recorded visits to the ED, 30.06% were injury related, 23.28% were due to psychiatric illness, 7.99% were alcohol related, 21.29% were drug related and 57.34% were due to a chronic medical condition	Average of 0.97 ED visits per person, per year
20	Feldman et al. 2018 [34]	2018	USA	To assess the prevalence of homelessness by gender	Retrospective survey from May 2015 to February 2016	Patients presenting to 3 EDs (a level trauma centre, a suburban hospital and an inner-city hospital)	309 (7%) out of 4395 unique participants were homeless			
21	Tsai et al. 2013 (b) [35]	2013	USA	To determine the ED use among homeless and domiciled VA service users	Retrospective cohort study using VA administrative workload databases from fiscal year 2010	Homeless and domiciled veterans presenting to the ED		64,099 (6.89%) out of 930,598 visits to the ED were made by homeless people		
22	Moulin et al. 2018 [36]	2018	USA	To determine the ED utilisation for patients with a primary mental health diagnosis	Retrospective analysis of OSHPD data from 2009 to 2014	Patients with a primary mental illness visiting acute care hospitals’ EDs		6153 (0.73%) out of 846,867 visits made to the ED by adult patients with mental illness were by homeless ED users		
23	Cheallaigh et al. 2017 [37]	2017	Ireland	To compare the use of unscheduled ED and inpatient care between housed and homeless patients	Observational cross-sectional study using electronic patient data in 2015	All ED visits and unscheduled admissions to one teaching hospital		2966 (6.3%) out of 47,174 ED attendances were made by homeless patients	Out of 2966 recorded visits to the ED, 7.6% presented for overdose and poisoning, 6.6% for alcohol-related issues, 5.6% for head injury, 4.8% for mental illness, 3.8% for abdominal pain and 2.9% for chest pain	Average of 3 ED visits per person per year and housed individuals had an average of 0.16 ED visits per, person per year
24	Yeniocak et al. 2017 [38]	2017	Turkey	To determine the sociodemographic and clinical characteristics of Turkish homeless patients who were brought to the ED by ambulance	Retrospective cross-sectional study from January to December 2014	Homeless adult patients brought to a Tertiary Training and Research Hospital by ambulance	167 (0.0835%) homeless patients attended the ED which serves an average of 200,000 patients each year		Out of 167 visits to the ED, 14.7% presented due to respiratory difficulty, 12.57% due to abdominal pain, 23.35% for clouded consciousness, 15.57% for generally impaired condition, 7.78% for traffic incidents and 5.39% for sharp object injury	
25	Lloyd et al. 2017 [39]	2017	Australia	To understand the profile and expressed needs of people seen by HEDLO in the ED in comparison to the general hospital population	Retrospective chart audit of data recorded in ED referral database and HEDLO files from October 2013 to January 2015	Homeless persons referred to HEDLOs in Queensland Health ED	117,996 presentations to the ED over 16-month period. Of these, 221 homeless people were referred to HEDLO		Out of 221 recorded visits to the ED, 25% presented due to mental health, 19% due to alcohol- and other drug-related issues, 39% for chronic medical conditions and 15% for social reasons	
26	Lombardi et al. 2019 [40]	2019	USA	To analyse national survey data to elucidate the differences between homeless and non-homeless patients’ ED visits	Retrospective study using NHAMCS dataset from 2005 to 2015	Patients presenting to non-federal hospital ED and outpatient departments		2750 (0.91%) out of 303, 326 visits to the ED were made by homeless persons	Out of 2750 recorded visits to the ED, 28.4% presented due to psychiatric diagnoses, (16.29% were not substance misuse related) 17.7% were drug use related, 1.2% were alcohol related, 1.78% were respiratory related and 1.09% were cardiovascular related	
27	Hastings et al. 2013 [41]	2013	USA	To determine predictors of repeat health service use in older veterans treated and released from the ED	Retrospective cohort study analysing VHA administrative datasets and the Vitals Mini File from 1 October 2007 to 30 June 2008	Patients aged 65 or over who were treated and released from a Veterans Affairs Medical Centre ED or urgent care clinic		374 (1.2%) out of 31,206 visits to the ED were made by homeless veterans		
28	Lam et al. 2016 [42]	2016	USA	To assesses the impact of homelessness on 30-day ED revisits and hospital readmissions among patients presenting with mental disorders	Secondary analysis of administrative data in the ED looking at visits made in 2012	Homeless patients presenting to the ED in an urban, safety-net hospital	4210 (4.6%) 0ut of 92,307 unique patients were homeless at any time during the study period	15,159 (10.9%) out of 139,414 visits to the ED were made by persons who were homeless at any time during the study period	Out of 15,159 recorded visits to the ED, 39.25% presentations were mental disorders and 60.75% were non-mental disorders	
29	Stenius-Ayoade. 2017 [43]	2017	Finland	To examine the role of mental disorders in relation to the use of primary healthcare services among homeless shelters in Helsinki	Retrospective analysis of electronic health records made by physicians and nurses working in primary health care from 2005 to 2008	Homeless persons in 4 shelters operating in the Helsinki metropolitan area			Out of 587 recorded visits to the Primary Health Care Emergency Rooms, 11% were for mental health and substance abuse, 38% were for trauma, 11% were for infections and 19% were for intoxications and convulsions	
30	Post et al. 2013 [44]	2013	USA	To determine the prevalence and types of ‘new media’ use among homeless patients who present to the ED	Observational cross-sectional survey from July to August 2012	Patients presenting to 3 urban, high-volume EDs in Connecticut		249 (4.3%) out of 5788 subjects enrolled in the study, reported episodes of homelessness in the past year.		
31	Moore et al. 2012 [45]	2012	Australia	To evaluate the accuracy of a predictive model to identify homeless people at risk of re-presentation to the ED	Prospective cohort study conducted from 1 April 2009 to 30 April 2009	Patients presenting to an adult, tertiary referral hospital ED, excluding those who died during study period	211 (7.31%) out of 2888 unique individuals who visited the ED were homeless	327 (9.92%) out of 3298 visits to the ED were made by homeless persons		
32	Doran et al. 2018 [46]	2018	USA	To characterise alcohol and drug use in a sample of homeless vs. non-homeless ED patients	Baseline survey interviews with patients at public hospital ED from November 2016 to September 2017	Random sample of patients who presented to an urban public hospital ED		316 (13.69%) out of 2309 patients were currently experiencing homelessness	Out of 316 recorded visits to the ED, 25% were substance use related	
33	Doran et al. 2013 [47]	2013	USA	To determine what multi-dimensional patient-level factors are most strongly associated with a 6-level gradient of VHA ED use	Cross-sectional analysis of data obtained from national VHA databases for fiscal year 2010	Veterans presenting to VHA ED services		64,091 (6.9%) out of 930,712 patients who visited the ED were homeless		
34	Ku et al. 2014 [48]	2014	USA	To examine the study characteristics and costs associated with homeless ED frequent users	Retrospective cross-sectional review of hospital and financial records for ED visits in 2006	Frequent users of the ED in an urban academic medical centre with a level 1 trauma and annual census of greater than 60,000 visits	74 (13.7%) out 542 frequent users were homeless	845 (15.5%) out of 5440 visits made by frequent users were made by homeless persons	Out of the 845 presentations to the ED, 12.9% were due to substance abuse, 10.9% were nervous system related, 8.9% were respiratory problems, 7.1% were cardiovascular problems and 8.3% were due to traumatic disorders	
35	Coe et al. 2015 [49]	2015	USA	To compare homeless patients’ utilisation of the urban ED in the USA with non-homeless patients	Cross-sectional study of the NHAMCS-ED electronic database for 2009 to 2010	Patients presenting to non-federal hospital ED and outpatient departments		1,302,256 (0.65%) out of 200,645,347 visits to the ED were made by homeless patients		
36	Amato et al. 2018 [50]	2018	USA	To compare emergency care utilisation between individuals with documented homelessness to those enrolled in Medicaid without documented homelessness	Retrospective cohort study using medical chart review between for the years 2013 and 2014	Patients presenting to a single, urban, academic, tertiary care centre		7532 (5.17%) out of 145,662 visits to the ED were made by persons with documented homelessness	Out of 7532 recorded visits to the ED, 20.1% of patients presented for mental health disorders, 13.4% were alcohol related, 1.12% were for drug overdose, 9.3% were for abdominal pain, 8.7% were for chest pain and 7.8% were for trauma	

*BHCHP*, Boston Health Care for the Homeless Program; *DSM*, Diagnostic and Statistical Manual; *ED*, emergency department; *HEDLO*, Homeless Emergency Department Liaison Officers; *DSM-IV*: *NEISS*, National Electronic Injury Surveillance System; *NHAMCS*, National Hospital Ambulatory Care Survey; *OSHPD*, California’s Office of Statewide Health Planning and Development; *VA*, Veteran Affairs; *VHA*, Veteran Health Affairs

### Quality assessment measuring risk of bias

Of the 36 included studies, only 8 studies received a score of 0 for all the risk of bias criteria. Risk of bias criteria were lacking in relation to generalisability of the study findings to the wider populations. This was often due to the study populations belonging to one or a few hospitals in a single city. Non-response bias was unclear where survey or interview methodologies were used to collect data (Table 2).

**Table 2:** Risk of bias assessment using BMJ quality assessment for prevalence studies

		Item 1	Item 2	Item 3	Item 4	Item 5	Item 6	Item 7	Item 8	Item 9	Item 10
Tadros et al. 2016 [15]	USA	0	0	0	0	0	0	0	0	0	0
Oates et al. 2009 [16]	USA	0	0	0	0	0	0	0	0	0	0
Holtyn et al. 2017 [17]	USA	1	1	0	1	0	0	0	0	0	3
Brown et al. 2010 [18]	UK	1	0	0	0	0	0	0	0	0	1
Cheung et al. 2015 [19]	Canada	1	0	0	0	0	0	0	0	1	2
Brown et al. 2013 [20]	USA	0	0	0	0	0	0	0	0	0	0
Raven et al. 2017 [21]	USA	1	1	0	1	0	0	0	0	1	4
Ku et al. 2010 [22]	USA	0	0	0	0	0	1	0	0	1	2
Feldman et al. 2017 [23]	USA	1	0	1	0	0	0	0	0	0	2
Jackson et al. 2019 [24]	USA	1	0	0	1	0	0	1	0	1	4
Lee et al. 2019 [25]	Australia	1	0	1	1	0	0	1	1	0	5
Tsai et al. 2013 (a) [26]	USA	0	0	0	0	0	0	1	0	0	1
Rodriguez et al. 2009 [27]	USA	1	0	1	1	0	0	0	0	0	3
Lin et al. 2015 [28]	USA	1	0	0	0	0	0	1	0	0	2
Mackelprang et al. 2014 [29]	USA	1	0	0	1	0	0	1	0	1	4
Doran et al. 2016 [30]	USA	1	0	0	1	0	0	0	0	0	2
Moore et al. 2011 [31]	Australia	0	0	0	0	0	0	0	0	0	0
Hammig et al. 2014 [32]	USA	0	0	0	0	0	0	0	0	0	0
Mackelprang et al. 2015 [33]	USA	1	0	0	0	0	0	1	0	0	2
Feldman et al. 2018 [34]	USA	1	0	1	0	0	0	0	0	0	2
Tsai et al. 2013 (b) [35]	USA	0	0	0	0	0	0	1	0	0	1
Moulin et al. 2018 [36]	USA	0	0	0	0	0	0	0	0	0	0
Cheallaigh et al. 2017 [37]	Ireland	0	0	0	1	0	0	0	0	0	1
Yeniocak et al. 2017 [38]	Turkey	1	0	0	0	0	0	1	0	0	2
Lloyd et al. 2017 [39]	Australia	1	0	0	0	0	1	1	0	1	4
Lombardi et al. 2019 [40]	USA	0	0	0	0	0	0	0	0	1	1
Hastings et al. 2013 [41]	USA	0	0	0	0	0	0	0	0	0	0
Lam et al. 2016 [42]	USA	1	0	0	0	0	0	0	0	0	1
Stenius-Ayoade. 2017 [43]	Finland	1	0		0	0	0	0	0	1	2
Post et al. 2013 [44]	USA	1	0	1	0	0	0	1	0	0	3
Moore et al. 2012 [45]	Australia	1	0	0	0	0	0	0	0	0	1
Doran et al. 2018 [46]	USA	1	0	0	1	0	0	0	0	0	2
Doran et al. 2013 [47]	USA	0	0	0	0	0	0	0	0	0	0
Ku et al. 2014 [48]	USA	1	0	1	0	0	0	1	0	0	3
Coe et al. 2015 [49]	USA	0	0	1	0	0	0	1	0	0	2
Amato et al. 2018 [50]	USA	1	0	0	0	0	0	1	0	0	2

**Item 1:** Was the study’s target population a close representation of the national population in relation to relevant variables, e.g. age, sex, occupation? **Item 2: **Was the sampling frame a true or close representation of the target population? **Item 3: **Was some form of random selection used to select the sample, or was a census undertaken? **Item 4:** Was the likelihood of non-response bias minimal? **Item 5:** Were data collected directly from the subjects (as opposed to a proxy)? **Item 6:** Was an acceptable case definition used in the study? **Item 7:** Was the study instrument that measured the parameter of interest (e.g. prevalence of low back pain) shown to have reliability and validity (if necessary)? **Item 8: **Was the same mode of data collection used for all subjects? **Item 9: **Were the numerator(s) and denominator(s) for the parameter of interest appropriate **Item 10: **Summary on the overall risk of study bias

### Overview of included studies and study populations

Twelve studies reported secondary analysis of existing national data sources, including the National Hospital Ambulatory Care Survey (NHAMCS) [15, 16, 20, 22, 32, 40, 49], National Electronic Injury Surveillance System (NEISS) [29], Veterans Affairs (VA) administrative data [26, 35] and Veterans Health Administration (VHA) databases [41, 47]. The extent of data overlap across studies which used similar databases could not be accurately estimated due to lack of clarity in the data inclusion criteria (Table 1). A further ten studies used retrospective analysis of secondary data sources focusing on smaller sub-populations such as one or a few hospital EDs [28, 33, 37–39, 42, 43, 45, 48, 50]. Six studies used a combination of both retrospective sampling techniques and prospective data collection, such as interviews or surveys [19, 21, 24, 34, 44, 46] and five studies prospectively interviewed or assessed patients presenting to the ED [17, 23, 27, 30, 31]. One study employed both a secondary analysis of retrospective data and prospective screening of a sample at one inner metropolitan hospital ED [25]. Some studies focused on only one presentation, such as injuries [29], as the cause of ED presentation by PEH (Table 1).

### Prevalence of homelessness in the ED

A total of 30 studies included data on the prevalence of homelessness in the ED, either reporting the proportion of unique patients who were experiencing homelessness or the proportion of ED visits made by PEH (Tables 3 and 4). Four studies reported both patient-level and visit-level data [31, 42, 45, 48]. The proportion of ED visits contributed by PEH ranged from 0.41% [15, 16] in two retrospective studies analysing NHAMCS data for 2005 to 19.6% [30] in a prospective study which assessed a random sample of patients presenting to an urban public hospital ED.

**Table 3:** Number and proportion of ED visits made by PEH

Study ID	Country	Study setting and population	Total number of ED visits during study period	Number of ED visits made by homeless persons	% of ED visits made by homeless persons
Lombardi et al. 2019 [40]	USA	Patients presenting to non-federal hospital ED and outpatient departments	303,326	2750	0.91
Moulin et al. 2018 [36]	USA	Patients with a primary mental illness visiting acute care hospitals’ EDs	846,867	6153	0.73
Doran et al. 2018 [46]	USA	Random sample of patients who presented to an urban public hospital ED	2309	316	13.69
Amato et al. 2018 [50]	USA	Patients presenting to a single, urban, academic, tertiary care centre	145,662	7532	5.17
Cheallaigh et al. 2017 [37]	Ireland	All ED visits and unscheduled admissions to one teaching hospital	47,174	2966	6.29
Tadros et al. 2016 (a) [15]	USA	Patients presenting to non-federal hospital ED and outpatient departments	124,043,357	679,854	0.55
Tadros et al. 2016 (b) [15]	USA	Patients presenting to non-federal hospital ED and outpatient departments	115,322,815	472,922	0.41
Doran et al. 2016 [30]	USA	Patients presenting to an urban public hospital ED	625	123	19.60
Lam et al. 2016 [42]	USA	Homeless patients presenting to the ED in an urban, safety-net hospital	139,414	15,159	10.87
Coe et al. 2015 [49]	USA	Patients presenting to non-federal hospital ED and outpatient departments	200,645,347	1,302,256	0.65
Hammig et al. 2014 [32]	USA	Patients presenting to non-federal hospital ED and outpatient departments	119,993,000	603,000	0.50
Brown et al. 2013 [20]	USA	Patients presenting to non-federal hospital ED and outpatient departments	480,000,000	2,808,000	0.59
Tsai et al. 2013 (a) [26]	USA	Homeless veterans presenting to VA EDs	930,712	64,091	6.89
Hastings et al. 2013 [41]	USA	Patients aged 65 or over who were treated and released from a Veterans Affairs Medical Centre ED or urgent care clinic	31,206	374	1.20
Post et al. 2013 [44]	USA	Patients presenting to 3 urban, high-volume EDs in Connecticut	5788	249	4.30
Doran et al. 2013 [47]	USA	Veterans presenting to VHA ED services	930,712	64,091	6.89
Moore et al. 2012 [31]	Australia	Patients presenting to a principal referral hospital ED	3,298	327	9.92
Moore et al. 2011 [45]	Australia	Patients presenting to an adult, tertiary referral hospital ED, excluding those who died during study period	64,177	6689	10.42
Brown et al. 2010 [18]	UK	Patients presenting to the Northern General Hospital ED and data from the Weston Park Weather Station	528,573	2930	0.55
Ku et al. 2010 [22]	USA	Patients presenting to non-federal hospital ED and outpatient departments	234,000,000	1,100,000	0.47
Oates et al. 2009 [16]	USA	Patients presenting to non-federal hospital ED and outpatient departments	115,322,815	472,922	0.41
Rodriguez et al. 2009 [27]	USA	Patients in the treatment areas of one urban hospital ED	50,172	9806	19.54

*ED*, emergency department; *VHA*, Veteran Health Affairs; *DSM*, Diagnostic and Statistical Manual

**Table 4:** Count of unique individuals experiencing homelessness in the ED

Study ID	Country	Study setting and population	Total number of unique patients who presented in the ED	Number of unique patients who were homeless	% of patients who were homeless
Lee et al. 2019 (b) [25]	Australia	Patients presenting to an inner metropolitan hospital ED in Melbourne	504	40	7.94
Feldman et al. 2017 [23] (also reported in Feldman et al. 2018 [34])	USA	Patients presenting to 3 EDs (a level trauma centre, a suburban hospital and an inner-city hospital)	4,395	309	7.03
Lam et al. 2016 [42]	USA	Homeless patients presenting to the ED in an urban, safety-net hospital	92,307	4210	4.56
Moore et al. 2012 [31]	Australia	Patients presenting to a principal referral hospital ED	2888	211	7.31
Moore et al. 2011 [45]	Australia	Patients presenting to an adult, tertiary referral hospital ED, excluding those who died during study period	40,942	1595	3.90

*ED* emergency department

Four studies focused on the utilisation of the ED by veterans experiencing homelessness [26, 35, 41, 47]. Three of these studies used national veterans’ affairs (VA) administrative data [26, 35, 41]. Two of these studies reported that veterans experiencing homelessness contributed to approximately 6.9% of all ED visits made by homeless persons [26, 35]. One study [51] found that homeless VA service users were approximately three times more likely to use the ED than domiciled VA service users.

### Mean number of visits to the ED by PEH in a year

**Fig. 2:**
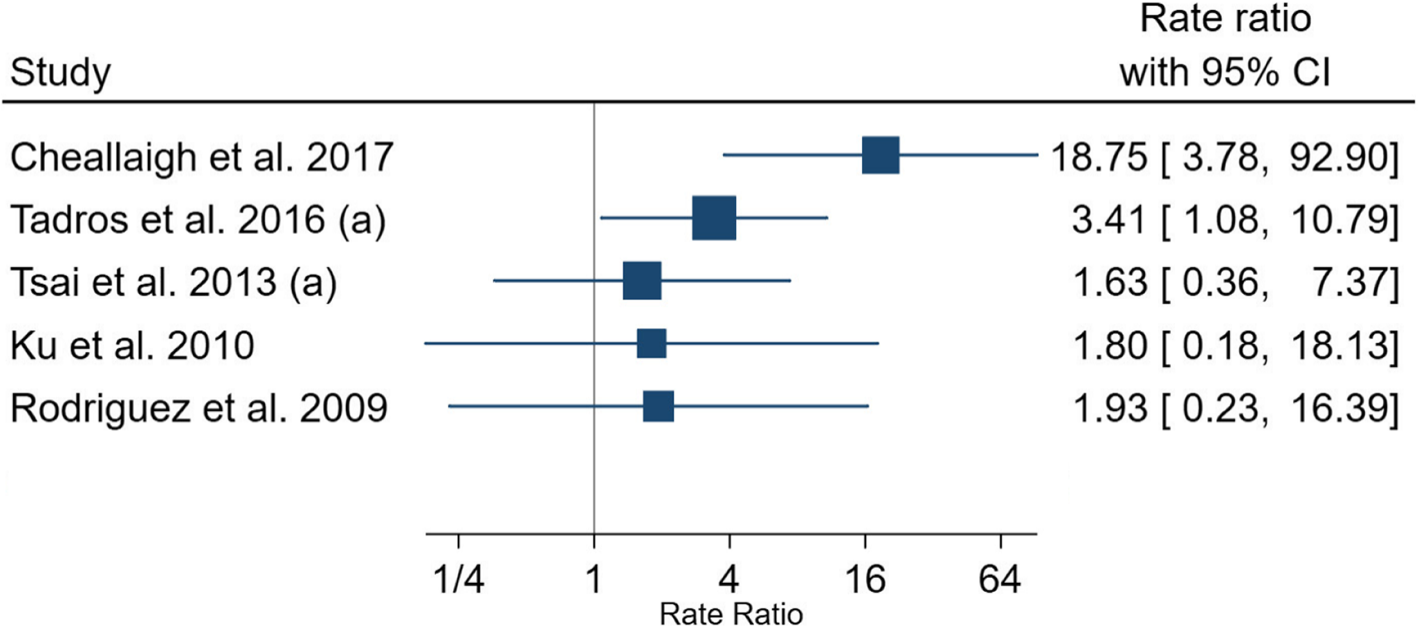
Rate ratio of number of ED visits per person, per year made by PEH compared with non-homeless populations. *ED*, emergency department; *PEH*, persons experiencing homelessness

Ten studies reported data on the mean number of visits to the ED per person, per annum, among the PEH. The value ranged from 0.72 visits to 5.8 visits per PEH, per year within the study period (Table 5). Five studies compared the mean number of visits to the ED between PEH and non-homeless populations, with the number of visits being consistently higher in the PEH compared with the non-homeless population [15, 22, 26, 27, 37]. Rate ratio ranged from 1.63 to 18.75 (Fig. 2). A study conducted in the USA also demonstrated that the proportion of ED visits contributed by PEH were rising at a faster pace than the non-homeless populations [15].

**Table 5:** Mean number of ED visits made by PEH in a year

Study ID	Country	Study setting and population	Sample size (***N***)	Mean number of ED visits per person per year	Std. Deviation	Follow-up time/study period
Holtyn et al. 2017 [17]	USA	Homeless, alcohol-dependent (met DSM-IV criteria) adults from an inpatient detoxification unit and homeless community agencies	86	4.4		26 weeks
Cheallaigh et al. 2017 [37]	Ireland	All ED visits and unscheduled admissions to one teaching hospital	2966	3		1 year
Tadros et al. 2016 (a) [15]	USA	Patients presenting to non-federal hospital ED and outpatient departments	679,854	5.8		5 years
Cheung et al. 2015 [19]	Canada	Homeless or precariously housed individuals who met criteria for a mental disorder with or without concurrent substance use dependence	3086	2.1		5 years, 6 months
Lin et al. 2015 [28]	USA	Homeless Medicaid recipients who received service from BHCHP	25,771	3.97		1 year
Mackelprang et al. 2015 [33]	USA	Patients presenting to the ED or inpatient departments of two urban teaching hospitals	1151	0.97		3 years
Tsai et al. 2013 (a) [26]	USA	Homeless veterans presenting to VA EDs	640,091	3.38	4.01	1 year
Moore et al. 2011 [45]	Australia	Patients presenting to an adult, tertiary referral hospital ED, excluding those who died during study period	6689	2.1		2 years
Ku et al. 2010 [22]	USA	Patients presenting to non-federal hospital ED and outpatient departments	550,000	0.72		2 years
Rodriguez et al. 2009 [27]	USA	Patients in the treatment areas of one urban hospital ED	191	5.8	2.2	14 weeks

*BHCHP*, Boston Health Care for the Homeless Program; *ED*, emergency department; *VA*, Veteran Affairs

### Reasons for presentation to the ED by PEH

Nineteen studies reported the primary reasons for presentation to the ED by PEH. Nine studies had a comparator group, providing the reasons for presentation to the ED among both PEH and non-homeless populations, allowing data comparisons [15, 22, 27, 29, 35, 37, 40, 48, 50].

The proportion of ED visits contributed by alcohol-related diagnoses ranged from 8% to 34% with four studies reporting a prevalence between 8.0% and 15.2%. The fifth study by Holtyn et al. [17] which reported 34% of visits contributed by alcohol-related diagnosis also used random breath collection in addition to self-reports. Among the two studies which compared homeless and non-homeless presentations, the relative risks (RR) ranged from 4.73 [50] to 6.83 [26].

The proportion of visits contributed by drugs, poisoning and substance misuse-related presentations ranged from 1.1% to 25%. Out of the three studies which compared PEH with non-homeless populations, RRs ranged from 1.05 [50] to 9.54 [26].

Injury-related diagnoses contributed between 7.8% and 55% of diagnoses. Among the two studies which compared the injury-related presentations between PEH and non-homeless populations, the RR ranged from 0.67 [50] to 1.55 [22].

The proportion of visits for pain or due to the need of analgesia ranged from 13% to 28%. Two studies which compared this data with non-homeless persons reported RRs of 0.92 [50] and 1.41 [26].

The proportion of ED visits attributed to non-substance misuse-related psychiatric and mental health-related conditions ranged from 5.8% to 36%. Out of the three studies which reported both homeless and non-homeless data, the RR ranged from 1.22 [22] to 4.42 [40]. One study using a veterans homeless population dataset showed that a high prevalence of psychiatric and mental health-related conditions contributed to the ED visits [26].

The proportion of patients presenting to the ED for cardiovascular conditions among the PEH ranged from 1.1% [40], in a study using national population data from the NHAMCS database, to 28% in a study utilising a homeless veteran dataset [26]. The RR, when comparing this value to non-homeless persons, ranged from 0.89 [26] to 1.03 [40].

Respiratory conditions contributed between 1.8% of ED attendance, in a study using national population data from the NHAMCS database, [40] to 15% in a study evaluating data of those brought by ambulance and non-trauma-related attendance [38]. Three studies reported both PEH and non-homeless data, producing RRs which ranged from 0.63 [40] to 1.01 [26].

### Deaths of PEH in the ED

**Fig. 3:**
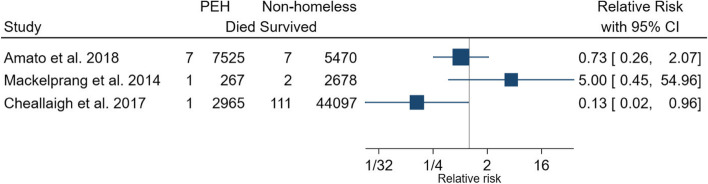
Relative risk of deaths in PEH attending the ED compared to non-homeless populations. *ED*, emergency department; *PEH*, persons experiencing homelessness

Four studies reported the number of homeless patients who died in the ED [29, 33, 37, 50]. The percentage of deaths reported by homeless persons in the ED ranged from 0.1% [37, 50] to 0.5% [33]. Three studies compared the proportion of homeless and non-homeless patients who died in the ED, producing RRs ranging from 0.13 [37] to 5.00 [29] (Fig. 3).

## Discussion

This study summarises the nature, extent and outcomes of presentations to the ED by PEH using systematic review methodology. PEH experience fragmentation of services, are often denied healthcare based on eligibility criteria and costs, and face stigma and discrimination at healthcare settings [12, 51–54]. Tailored services, including outreach-based interventions that are able to deliver primary healthcare to patients’ temporary residence or in the urban streets where they frequent, are likely to bring positive changes and minimise the need for ED visits [55]. Such outreach services can also minimise physical and disability-related barriers to accessing primary healthcare.

Injury-related diagnoses were one of the most common reasons for presentation to the ED among PEH. Consistent with previous findings, PEH suffer a disproportionate burden of injuries compared to non-homeless persons [32]. Mental health and psychiatric-related diagnoses were identified as another important primary reason for presentation to the ED. Psychiatric diagnoses were particularly prevalent in homeless veterans [26]. A previous study has shown than severe mental health is more prevalent in veteran populations than non-veteran populations [56].

Only four papers reported on the number of deaths among PEH in the ED. Recent literature has reported that a very high proportion of PEH leave the ED before being treated [51]. Further research is required to obtain a more accurate comparison of the death rates in the ED between PEH and the general population. This comparison may provide useful insights regarding the severity of health conditions when PEH present to the ED and offer a comparison between the standard of care received by PEH in the ED versus the non-homeless population.

This study has illustrated that injury-, mental health- and substance misuse-related health conditions dominate the reasons for presentations to the ED by PEH. This highlights the importance of factoring homelessness into the ED triage prioritisation process to improve patient outcomes. There is a continued need to improve the provision and implementation of mental health- and psychiatric-related support in the community. Furthermore, ED service providers should work closely with primary healthcare services to break down barriers to accessing healthcare among homeless populations. PEH are known to be less likely to be registered with a mainstream general practice compared with the general population. Although specialist primary healthcare centres for homeless persons have been established in an attempt to address such disparities, there is a need for the mainstream services to be more inclusive of homeless populations [54].

The COVID-19 pandemic has resulted in job losses and increases in domestic violence which is likely to result in a rise in homelessness. Therefore, public services must identify those who are in an unstable housing situation and assist them before they are pushed into homelessness [57, 58]. Innovative methods of support offered to PEH during the pandemic need to be sustained, for example emergency housing and the use of technology-assisted methods of counselling and communication, [59]. Strengthening primary care, including specialist homelessness services [60], community pharmacy [61], and enabling ED personnel to triage and treat PEH for overlapping health conditions, is imperative to prevent ill health and promote outcomes when they present to the ED. Clinical guidelines need to be further inclusive of multi-morbidity, including dual diagnosis of substance misuse and mental health, to prevent and mitigate the impact of homelessness on health [62]. Further research should include outreach-based innovative and integrated interventions offering preventative services and healthcare that can promote health, offer early diagnoses and treatment, and minimise ED attendance [63, 64].

### Limitations

This systematic review has some limitations. Homelessness status is often based upon self-reported data [15, 51]. In addition, PEH who reside in temporary shelters such as emergency accommodation, hostels or charity services may use corresponding addresses when presenting to the ED. Therefore, within the included studies, it is likely that street dwellers are more commonly identified as PEH in the ED records compared with patients experiencing other forms of homelessness. Many patients may also be using the postcode of their last permanent domicile when presenting to health services. As a result, the numbers presented in the literature likely underestimate the actual number of attendances made by PEH. The definition of homelessness also varies between countries and study settings. In addition, psychiatric-, substance misuse-, alcohol- and injury-related presentations often overlap when reporting primary reasons for presentation to the ED. Therefore, it may be useful to apply specific classification methods to record the primary reason for presentation in order to prevent such overlap. Data overlap was observed across studies included in this review which used the same database for the study purpose. Furthermore, some of the studies relied on self-reported data gathered through interviews [52]. In addition, the included studies represented a small number of countries where the studies were conducted.

## Conclusions

Drug-, alcohol- and injury-related presentations dominate the reasons for ED visits by PEH. Wide variations in the data were observed in regard to attendance and treatment outcomes. There is a need for an integrated discharge and referral pathway between ED and primary health, housing and social care to minimise frequent usage and improve attendance outcomes.

## Supplementary Information

**Additional file 1.** PRISMA Checklist (contains completed checklist).

**Additional file 2.** Example Search strategy.

## Data Availability

The datasets generated during and/or analysed during the current study are available from the corresponding author on reasonable request.

## Abbreviations

PEH: Persons experiencing homelessness
ED: Emergency department
HUD: Housing and Urban Development
NHAMCS: National Hospital Ambulatory Care Survey
NEISS: National Electronic Injury Surveillance System
VA: Veterans Affairs
VHA: Veterans Health Administration
RR: Relative risk
