# Supplementary material for: Homelessness and the use of Emergency Department as a source of healthcare: a systematic review
Source: Int J Emerg Med. 2022 Jul 28;15:32. doi: 10.1186/s12245-022-00435-3 (PMC9330962; doi:10.1186/s12245-022-00435-3)
Supplement: Supplementary file 2 — Additional file 2. Example Search strategy. [file 12245_2022_435_MOESM2_ESM.docx]

**Electronic Supplement 2: Example search strategy**

Database: Ovid MEDLINE(R) <1946 to September Week 5 2020>

Search Strategy:

--------------------------------------------------------------------------------

1 Homeless Youth/ or Homeless Persons/ or homeless.mp.

2 homelessness.mp.

3 no fixed abode.mp.

4 rough sleeper$.mp.

5 street dweller$.mp.

6 1 or 2 or 3 or 4 or 5

7 emergency department$.mp. or Emergency Service, Hospital/

8 (accident and emergency).mp. [mp=title, abstract, original title, name of substance word, subject heading word, floating sub-heading word, keyword heading word, organism supplementary concept word, protocol supplementary concept word, rare disease supplementary concept word, unique identifier, synonyms]

9 urgent care.mp.

10 7 or 8 or 9

11 6 and 10

12 limit 11 to (English language)

***************************
